# Lactation stage impacts the glycolytic function of bovine CD4^+^ T cells during *ex vivo* activation

**DOI:** 10.1038/s41598-020-60691-2

**Published:** 2020-03-04

**Authors:** Jordan M. Eder, Patrick J. Gorden, John D. Lippolis, Timothy A. Reinhardt, Randy E. Sacco

**Affiliations:** 1 Immunobiology Interdepartmental Graduate Program, Iowa State University, Ames, IA United States; 2 Veterinary Diagnostic and Production Animal Medicine, Iowa State University, Ames, IA United States; 3 Ruminant Diseases and Immunology Research Unit, National Animal Disease Center, USDA, Agriculture Research Service, Ames, IA United States

**Keywords:** Adaptive immunity, Lymphocytes

## Abstract

Dairy cattle undergo dynamic physiological changes over the course of a full lactation into the dry period, which impacts their immunocompetence. During activation, T cells undergo a characteristic rewiring to increase the uptake of glucose and metabolically reprogram to favor aerobic glycolysis over oxidative phosphorylation. To date it remains to be completely elucidated how the altered energetic demands associated with lactation in dairy cows impacts T cell metabolic reprogramming. Thus, in our *ex vivo* studies we have examined the influence of stage of lactation (early lactation into the dry period) on cellular metabolism in activated bovine CD4^+^ T cells. Results showed higher rates of glycolytic function in activated CD4^+^ T cells from late lactation and dry cows compared to cells from early and mid-lactation cows. Similarly, protein and mRNA expression of cytokines were higher in CD4^+^ T cells from dry cows than CD4^+^ T cells from lactating cows. The data suggest CD4^+^ T cells from lactating cows have an altered metabolic responsiveness that could impact the immunocompetence of these animals, particularly those in early lactation, and increase their susceptibility to infection.

## Introduction

Physiological stresses of pregnancy and lactation are energetically demanding, with glucose being shuttled to mammary glands and/or fetus depending on lactation stage and gestation^1^. Though ruminants rely heavily on volatile fatty acids, specifically acetate and propionate for energy in whole-body physiology, bovine lymphocytes rely on glucose as their major energy source. During lactation, cells are in competition for glucose with the mammary gland, as it is required for lactose synthesis^2,3^. Like other species, increased glucose uptake is necessary during bovine T cell activation^4,5^.

Previous research shows metabolic reprogramming occurring within T cells during activation. Metabolic reprogramming refers to “the Warburg effect”, a phenomenon first described in cancer cells, which utilize aerobic glycolysis in oxygen-rich environments^6^. This phenomenon has since been reported in many immune cells, including T cells^7–10^. CD4^+^ T cells in a resting state utilize oxidative phosphorylation, but with engagement of the T cell receptor (TCR) and CD28, glucose uptake is increased and downstream signaling within the cell occurs^4,11^. This metabolic switch enables cells to utilize mitochondrial respiration, i.e. oxidative phosphorylation (OXPHOS) to upregulate mitochondrial biogenesis during activation to increase the generation of biomolecules necessary for clonal expansion^12,13^ while simultaneously increasing the glycolytic rate of the cell. An increase in glycolysis is essential to cell growth and proliferation^4^. Further, aerobic glycolysis regulates immunity by means of glycolytic enzymes; for example, GAPDH, has a role as an RNA-binding protein to regulate post-transcriptional expression of cytokines, like IFN-γ^14^.

How T cells from cattle in different lactation stages or in dry cows metabolically reprogram is not yet understood. Shedding some light, Schwarm *et al*.^15^ showed activated peripheral blood cells (PBMCs) from cows prepartum and postpartum have altered levels of oxygen consumption rate (OCR). PBMCs exhibit high OCR at two and five weeks postpartum, suggesting activation may be modulated by energy and nutrient availability and those with a negative energy balance may have resultant compromised immunity^15^.

Early lactating cows are known to be susceptible to infections and metabolic disorders. Cows in this stage experience greater metabolic stress than cows later in lactation due to a homeorhetic shift to support lactogenesis^16,17^. Typically, cows in early lactation exhibit negative energy balance caused by insufficient feed intake necessary to meet the increased energetic and calcium demands of milk production^18,19^. In efforts to provide an alternative energy source during the increased demands of milk production, the early lactating cow must draw on fat and protein stores^20^. While glucose is increased in the dairy cow by hepatic gluconeogenesis, glucose is used for milk synthesis and not body tissues^21^. Adipocytes switch from lipogenesis to lipolysis and release lipids into the bloodstream for lactogenesis, which may lead to subclinical ketosis^20,22–24^. At the same time, the cow very often suffers from subclinical hypocalcemia due to diet constraints and slow mobilization of calcium stores. Ultimately, the dairy cow in early lactation is faced with major metabolic stressors, high blood levels of non-esterified fatty acids and marginal blood calcium. Additionally, increased plasma lipids may further impair immunity^25,26^.

During lipid mobilization, non-esterified fatty acids (NEFAs), like saturated fatty acids palmitic and stearic acid, are highest in blood, as well as phospholipids^25,27^. An increase in saturated fatty acids in plasma leads to incorporation into the cellular membrane, which has been suggested to negatively affect immune function^25^. It has been proposed in T cells, free fatty acids, such as palmitic and stearic acid, at a low concentration enter the cell and aid in “proliferation, cytokine production and lactate production,” but a high concentration has negative impacts leading to mitochondrial dysfunction and apoptosis^26,28^.

Adding to increased metabolic stress, cows may be predisposed to new infections during the late dry period, which can consequently develop into clinical presentation in the first weeks of lactation^18,29,30^. It is suggested that alterations in immune function in early lactation cows is caused by high concentration of NEFAs and ketone bodies in the plasma due to increased lipid mobilization^18,27,31,32^.

Alternatively, mid-lactation cows have been categorized as immunocompetent, as balance between milk production and energy intake is restored. In a study by Shuster *et al*.^33^, mid-lactation cows are better protected during an *E. coli* infection than early lactation cows. As cows move into late lactation, milk production is decreased though their feed intake is maintained. Energy is diverted to the growing fetus instead of the mammary glands^24^. At this time, cows in late lactation experience a shift in T cell function, polarizing toward Th2 when activated in the second trimester of pregnancy^34^. Upon entering the dry and pre-transition period cows are no longer lactating and T cell polarization, upon activation, skews back toward a Th1 profile, exhibiting a pro-inflammatory phenotype to support the delivery of the newborn^34^. The stress caused by initiation of lactation has been implicated in temporary immunosuppression accompanied by a Th2-dominant T cell profile exhibited in early lactation^30^. Thus, as cows transition from the dry period to lactation, there are differences in the directionality of Th bias.

There is very little information examining cellular immunometabolism in cattle. This is especially true regarding CD4^+^ T cells, which are crucial in protecting against pathogens, as both effector and memory populations. During each lactation stage and the dry period, the immune system is likely to have varying degrees of function and compensatory mechanisms for protection. In this study we expand on the work from Schwarm *et al*.^15^ to determine the effect lactation has on function and metabolic reprogramming in *ex vivo* activated CD4^+^ T cells.

## Results

### Serum components establish energy balance in dairy cows

**Figure 1:**
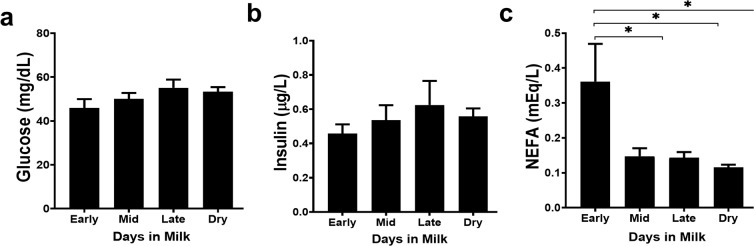
Glucose, insulin, and non-esterified fatty acids concentrations were determined from serum samples from dairy cows from different lactation stages and dry cows. Cows were separated into groups according to lactation stage as determined by days in milk (DIM) or indicated as dry for those not lactating. Early lactation cows (n = 5) were 14–43 DIM, mid lactation cows (n = 6) were 81–147 DIM, late lactation cows (n = 6) were 243–354 DIM, and dry cows are not lactating. Glucose and NEFAs were analyzed by colorimetric assay and insulin was analyzed by using an ELISA. Data shown are mean ± SEM. One-way ANOVA with Sidak’s multiple comparisons among all stages. *p < 0.05.

Because energy balance is important in understanding physiology of lactation stage, we analyzed serum glucose, insulin, and NEFA levels from cows in each stage. No differences in glucose levels were observed among any lactation stage (Fig. 1a). Insulin increased slightly from early lactation to late lactation, then decreased slightly in dry cows (Fig. 1b). Lastly, during early lactation cows are commonly in negative energy balance as they are unable to consume enough feed to meet energy demands of lactation. Thus, lipids are mobilized and NEFA concentrations are elevated. In Fig. 1c, as predicted, we show that early lactation cows have a significantly higher NEFA concentration than cows in later stages (*p < 0.05).

### Metabolic reprogramming occurs during activation of bovine CD4^+^ T cells

Quiescent CD4^+^ T cells predominantly depend on OXPHOS to support cellular functions. However, upon activation, CD4^+^ T cells undergo metabolic reprogramming. Aerobic glycolysis is then increased, and at a greater capacity than mitochondrial respiration to support rapid ATP generation and production of metabolic intermediates needed to support cell cycle progression and proliferation. To determine whether CD4^+^ T cells from ruminants have the same metabolic shift as activated CD4^+^ T cells in nonruminant species, and further, to determine whether metabolic reprogramming is impacted by stage of lactation, bovine CD4^+^ T cells were stimulated with plate-bound anti-CD3 and soluble anti-CD28 for 24 hours. Cellular activation was confirmed by flow cytometric analyses. Stimulated cells increased in size as measured by forward scatter in comparison to unstimulated cells (data not shown).

**Figure 2:**
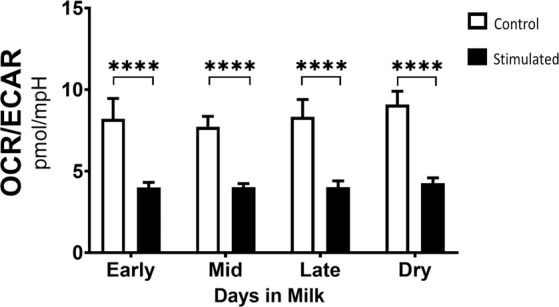
Metabolic reprogramming was observed in activated CD4^+^ T cells from dairy cows. (a) Peripheral blood CD4^+^ T cells were sorted and activated for 24 hours with plate-bound CD3 (5 μg/ml) and soluble CD28 (1 μg/ml) mAb and their metabolic phenotype was analyzed using the Seahorse extracellular flux analyzer. The OCR/ECAR ratio was measured from stimulated (black bars) and control T cells (white bars) in each lactation stages- early (14–43 DIM, n = 5), mid (81–147 DIM, n = 6), late (243–354 DIM, n = 6), dry (not lactating, n = 6). Data were log-transformed and shown are mean ± SEM. P values of CD4^+^ T cells were calculated by ordinary two-way ANOVA and Sidak’s multiple comparison post-hoc. ****p < 0.001.

After stimulation, we assessed metabolic switch by analyzing the ratio of Oxygen Consumption Rate (OCR) as a measurement of mitochondrial respiration to Extracellular Acidification Rate (ECAR) as a measurement of glycolysis and compared that to unstimulated cells. Stimulated bovine CD4^+^ T cells show a decrease in OCR/ECAR, indicative of the reported reprogramming favoring aerobic glycolysis seen in CD4^+^ T cells from nonruminant species (Fig. 2). Unstimulated, control cells had a higher OCR/ECAR ratio, indicative of being in a resting state and favoring OXPHOS (Fig. 2). Across all stages of lactation and in the dry period, activated bovine CD4^+^ T cells metabolically reprogrammed to favor aerobic glycolysis, exhibited by the shift in the OCR/ECAR ratio between resting and activated states. There were no significant differences in OCR/ECAR ratio among lactation stages and the dry period.

### Activation-induced changes in bovine CD4^+^ T cell mitochondrial respiration

**Figure 3:**
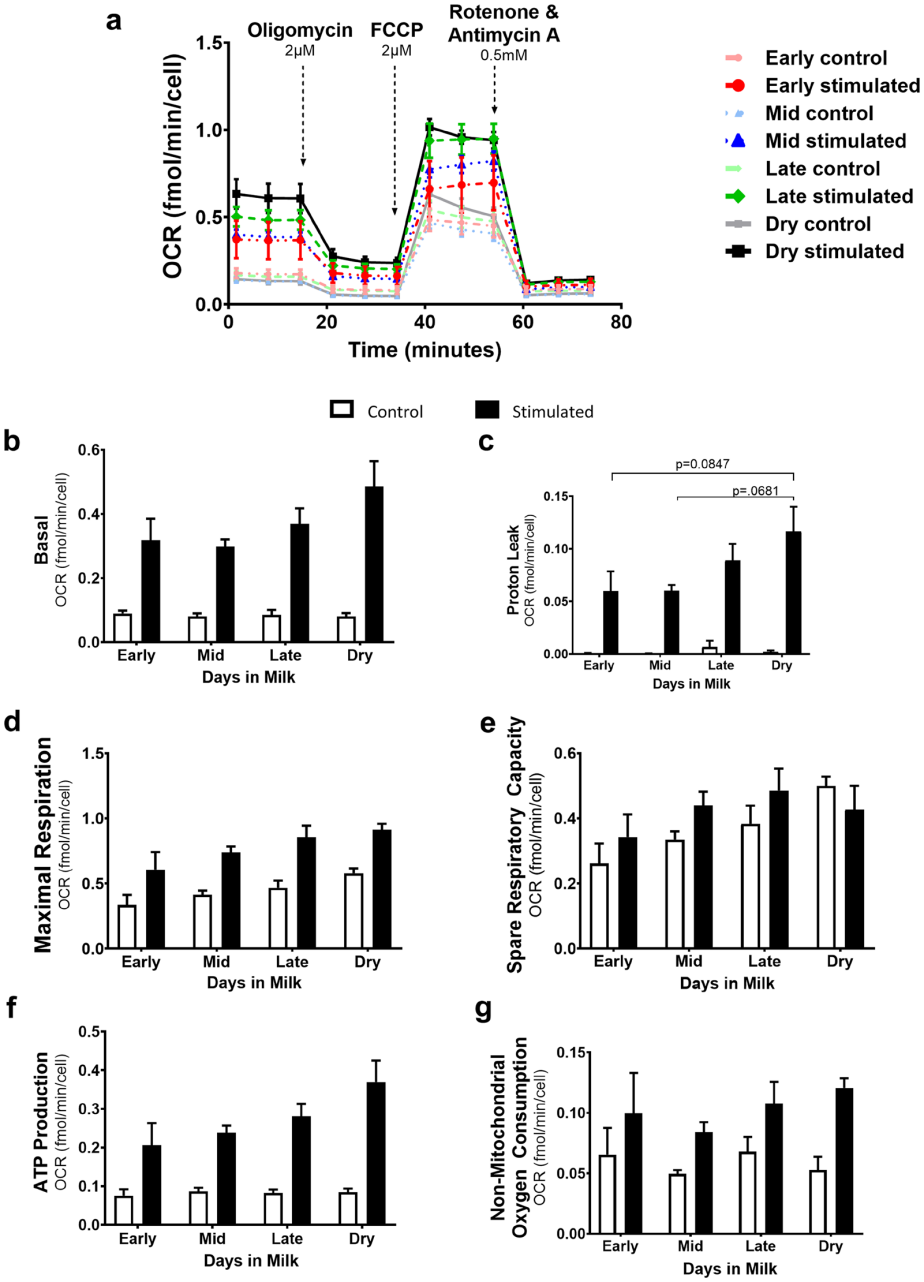
Mitochondrial function of peripheral blood CD4^+^ T cells from dairy cows of different lactation stages was assessed. Peripheral blood CD4^+^ T cells were sorted and activated with plate-bound CD3 (5 μg/ml) and soluble CD28 (1 μg/ml) mAb for 24 hours and mitochondria function was analyzed using the XF Cell Mito Stress Test kit (black lines/bars). Lighter lines/white bars represent control, unstimulated cells cultured for 24 hours, used as a point of reference for stimulated CD4^+^ T cells (darker lines/black bars) from cows in different stages of lactation and dry cows. Lactation stages were assigned as the following: early (14–43 DIM, n = 5), mid (81–147 DIM, n = 6), late (243–354 DIM, n = 6), dry (not lactating, n = 6). (**a**) Mitochondria function kinetics were recorded in real-time measuring oxygen consumption rate (OCR) under basal conditions and in response to electron transport chain inhibitors oligomycin (complex V), FCCP (a protonophore), and rotenone and antimycin A (complex I and complex III). This was used to calculate the following parameters: (**b**) Basal OCR, (**c**) Proton Leak, (**d**) Maximal Respiration, (**e**) Spare Respiratory Capacity, and (**f**) ATP Production. Data shown are mean ± SEM. Mitochondrial stress test was analyzed by Kruskal-Willis and Dunn test for multiple comparisons, post-hoc. Adjusted p-values were reported using Benjamini-Hochberg multiple comparison correction and false discover rate (FDR). *p < 0.05. FCCP, carbonyl cyanide-4-(trifluoromethoxy) phenylhydrazone.

To further characterize metabolic reprogramming within activated bovine CD4^+^ T cells, we isolated cells from dairy cattle at differing lactation stages and the dry period to evaluate the effects of activation on mitochondrial respiration (Fig. 3a). Interrogating different complexes of the mitochondrial electron transport chain (ETC) by injecting targeted inhibitors, allowed us to observe changes in mitochondrial function between unstimulated and stimulated bovine CD4^+^ T cells. Despite the metabolic shift to aerobic glycolysis, activated bovine CD4^+^T cells increased mitochondrial respiration as well, a trait conserved across species. Our results show stimulated CD4^+^ T cells from cows later in lactation and gestation tended to exhibit higher basal OCR (Fig. 3b) and proton leakage (Fig. 3c) than those in early and mid-lactation, but the differences were not statistically significant. ATP production by activated CD4^+^ T cells (Fig. 3f), as measured by changes in OCR, also tended to be higher relative to the progression of lactation and into the dry period. Similarly, we show consistent maximal respiration (Fig. 3d) and spare respiratory capacity (Fig. 3e) among stages. Overall, while late lactation and dry cows tended to show higher levels of OCR, mitochondrial respiration was not significantly different in activated CD4^+^ T cells from each lactation stage.

### Mitochondrial respiration changes driven by increased mitochondrial mass in bovine CD4^+^ T cells

**Figure 4:**
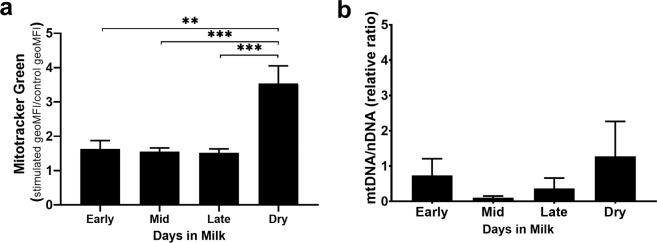
Mitochondria mass assessed as part of functional differences of activated bovine CD4^+^ T cells. Peripheral blood CD4^+^ T cells from dairy cows were stimulated *ex vivo* for 24 hours with plate-bound CD3 (5 μg/ml) and soluble CD28 (1 μg/ml) mAb. Cells were stained with (**a**) Mitotracker green (25 nM) to determine mitochondrial mass by flow cytometry. Data represents geometric MFI (geoMFI) ratio of stimulated to unstimulated CD4^+^ T cells from each lactation stage, early (14–43 DIM, n = 5), mid (81–147 DIM, n = 6), late (243–354 DIM, n = 6), dry (not lactating, n = 6). (**b**) qPCR analysis of relative mitochondrial DNA to nuclear DNA ratio (mtDNA/nDNA). Data presented are mean ± SEM. Ratio of Mitotracker green geoMFI was analyzed with one-way ANOVA using Tukey’s multiple comparison of CD4^+^ T cells among all stages. mtDNA/nDNA was analyzed with Brown-Forsythe ANOVA with Dunnett T3 multiple comparison test **p < 0.01, ***p < 0.001.

To further study potential reasons for increased mitochondrial respiration, more apparent in late lactation and dry cows, we investigated changes in mitochondrial mass using Mitotracker green in stimulated and unstimulated bovine CD4^+^ T cells. We then compared geometric mean fluorescent intensity (geoMFI) between all lactation groups and dry cows by the ratio of stimulated CD4^+^ T cells to unstimulated CD4^+^ T cells and were able to determine the increase in mitochondrial mass in each stage of lactation and dry cows (Fig. 4a). Early, mid, and late lactation groups had a ~1.5-fold increase in mitochondrial mass compared to unstimulated cells. CD4^+^ T cells from dry cows have over a 3.5-fold increase in mitochondria mass compared to unstimulated cells. Increased mitochondrial mass seen in CD4^+^ T cells from dry cows was significantly higher than that of stimulated CD4^+^ T cells from early (**p < 0.01) as well as from mid and late lactation (***p < 0.001). To determine whether the increase in mass was biogenesis-related, we quantified the ratio of mitochondrial DNA to nuclear DNA (mtDNA/nDNA) (Fig. 4b). There were no significant differences in mtDNA/nDNA among CD4^+^ T cells from any lactation group. Taken together, activated CD4^+^ T cells from dry cows have a greater increase in mitochondrial mass than lactating cows.

### Bovine CD4^+^ T cell glycolysis rate is altered during lactation

**Figure 5:**
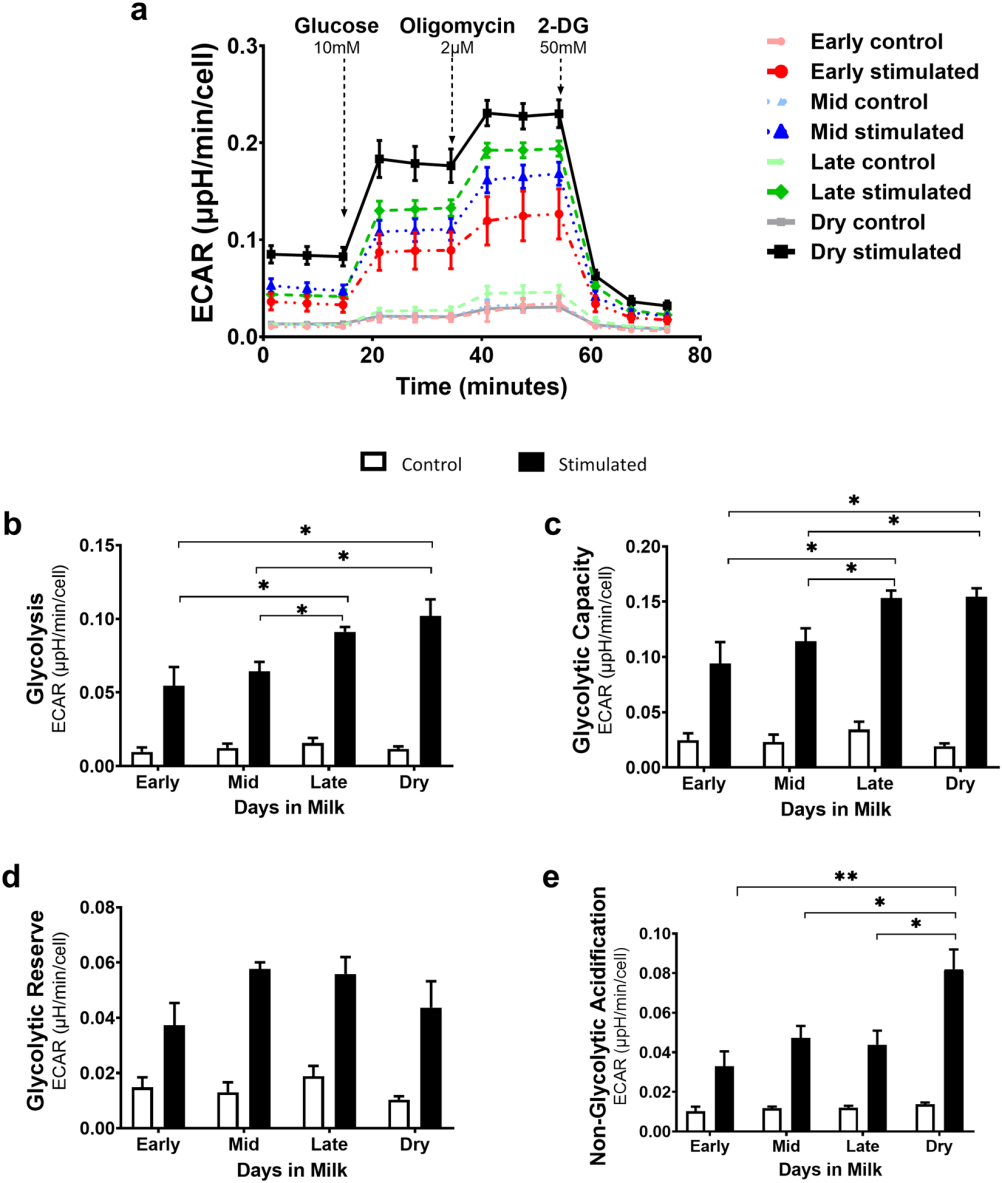
Metabolic shift towards aerobic glycolysis occurs in activated bovine CD4^+^ T cells. The glycolytic function was assessed using the XF Glycolysis Stress Test kit on bovine CD4^+^ T cells isolated from peripheral blood. Cells were activated *ex vivo* for 24 hours with plate-bound CD3 (5 μg/ml) and soluble CD28 (1 μg/ml) mAb. Stimulated cells are represented by darker lines/black bars. Control, unstimulated cells are represented by lighter lines/white bar and were cultured for 24 h. Extracellular acidification rate (ECAR) was recorded in real-time showing glycolytic function in a kinetic graph. (**a**) Cells were starved of glucose for 1 hour, the first injection of 10 mM glucose measures (**b**) the rate of glycolysis. Other parameters measured include (**c**) glycolytic capacity, (**d**) glycolytic reserve and (**e**) non-glycolytic acidification. Data presented are mean ± SEM. Glycolytic stress test was analyzed by Kruskal-Willis and Dunn test for multiple comparisons, post-hoc. Adjusted p-values were reported using Benjamini-Hochberg multiple comparison correction and false discover rate (FDR). *p < 0.05. Note: Oligomycin stimulates maximum ECAR by inhibiting ATP synthase. 2-deoxyglucose (2-DG) inhibits glycolysis and provides baseline ECAR.

After observing the expected reprogramming in activated bovine CD4^+^ T cells (Fig. 2a), we evaluated the change in glycolytic function. To examine glycolytic function, cells were stimulated for 24 hours in complete media, starved of glucose for one hour, and then injected with a saturating amount of glucose in order to assess glycolytic rate in an energy replete environment. Stimulated bovine CD4^+^ T cells across all lactation stages markedly increased their glycolytic rate compared to unstimulated cells. Notably, stimulated CD4^+^ T cells from dry cows and late lactation cows had a higher glycolytic rate (p < 0.05*) (Fig. 5a,b) and an increased ability to utilize glycolysis at a higher capacity (p < 0.05*) (Fig. 5c) than stimulated CD4^+^ T cells from cows in early and mid-lactation. Despite later stages having a more rapid flux in glycolysis, all stages had a similar glycolytic reserve (Fig. 5d), implying CD4^+^ T cells from all stages function at similar levels relative to their theoretical maximum during an increased energetic demand. Dry cows also had a higher level of non-glycolytic acidification (Fig. 5e) than lactating cows. To summarize, CD4 ^+^ T cells isolated from cows throughout each stage of a full lactation cycle increase their glycolytic rate and their overall capacity to support cellular function primarily through glycolysis, in the absence of significant mitochondrial respiration. Our data further suggest cells from cows in varying lactation stages have a similar glycolytic reserve, which may be relative to the activity of glycolytic machinery employed during activation.

### Metabolic enzyme mRNA expression is not reflective of metabolic status

**Figure 6:**
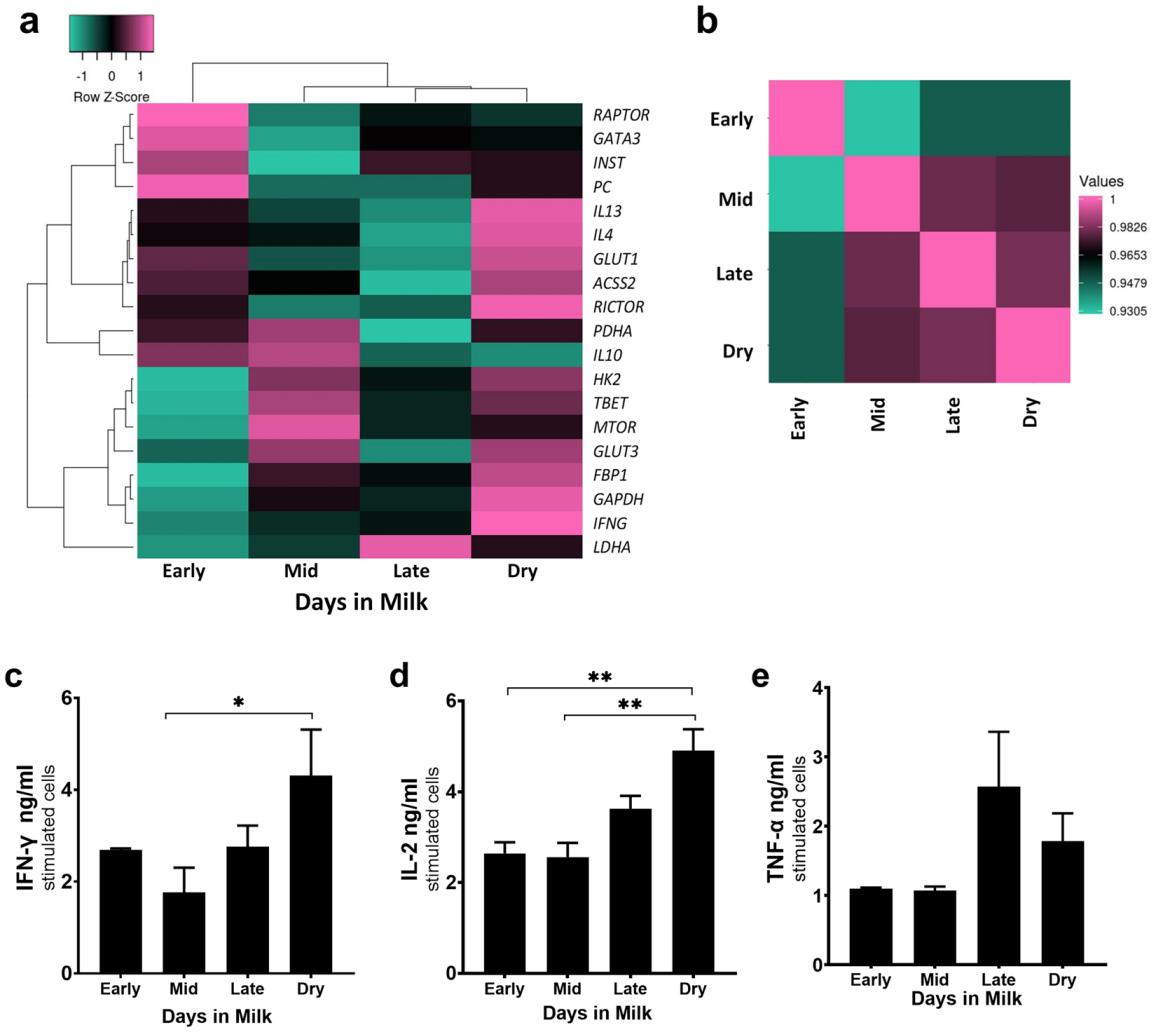
mRNA expression of enzymes and proteins involved in the immune response differ among lactation group. Peripheral blood CD4^+^ T cells were sorted and activated for 24 hours with plate-bound CD3 (5 μg/ml) and soluble CD28 (1 μg/ml) mAb. qPCR relative expression is the log2 of stimulated cells to unstimulated cells. (**a**) Expression-based heatmap of 19 genes involved in eliciting an immune response were analyzed and hierarchically clustered by average linking and Pearson’s distance measurement by the log2 relative expression from qPCR. (**b**) Pairwise Pearson’s correlation plot of the lactation groups (Pearson correlation coefficient 0.9305–1 for all lactation stages). Supernatants from unstimulated cells and cells stimulated for 24 hours with anti-CD3:anti-CD28 were used in ELISAs detecting the following cytokines (**c**) IFN-γ, (**d**) IL-2, (**e**) TNF-α. Unstimulated cells were below the level of detection in all ELISAs. Data presented are the mean ± SEM. One-way ANOVA with Tukey’s multiple comparison of CD4^+^ T cells among all stages. *p < 0.05, **p < 0.01.

Aerobic glycolysis has been shown to play a role in the regulation of immunity using enzymes involved in glycolysis as RNA-binding proteins to control translation of certain cytokines or inhibit T cell effector function^14,35^. We examined mRNA expression of genes involved in glycolysis, signaling molecules with roles in glucose metabolism, cytokine production, and proliferation, as well as cytokine signaling. The heatmap is indicative of relative expression of genes of interest from stimulated and unstimulated bovine CD4^+^ T cells (Fig. 6a). Metabolic genes of interest included *GAPDH*, hexokinase II *(HK2), LDHA*, and *TBET*. Previous data shows CD4^+^ T cells isolated from dry cows are highly glycolytic (Figs. 2 and 5) and here we show mRNA expression of glycolytic genes and regulators of IFN-γ increased in CD4^+^ T cells from dry cows compared to CD4^+^ Τ cells from the other lactation stages. Interestingly, CD4^+^ Τ cells from mid-lactation cows expressed similar mRNA levels of *TBET* and *HK2* as cells from dry cows, but slightly less *GAPDH* and lower *LDHA* (Fig. 6a). mRNA expression of CD4 ^+^ Τ cells from late lactation show an increased *LDHA*, and moderate expression of all glycolytic and IFN-γ regulating genes. Remarkably different from cells of other lactation stages and dry cows, CD4^+^ T cells from early lactation cows have depressed mRNA expression of all glycolytic genes and those related to production of IFN-γ.

### Gene expression of signaling molecules upstream from aerobic glycolysis is generally enhanced in CD4^+^ T cells isolated from early lactation cows

Additionally, we examined gene expression of molecules involved in signaling during activation, such as insulin receptor *(INSR)*, GATA3, Raptor *(RAPTOR)*, and *MTOR*. mRNA expression of molecules involved in signaling and promoting aerobic glycolysis directly or as a transcription factor regulator, *(INSR, GATA3, RAPTOR)* are more highly expressed in CD4^+^ T cells from early lactation cows. In contrast to other signaling molecules involved upstream of aerobic glycolysis, *MTOR* has a similar expression pattern as *HK2*. CD4^+^ T cells isolated from dry or mid-lactation cows express *MTOR* at a higher level than cells from late lactation, whereas cells from early lactation cows exhibit lowest expression.

### Pearson’s pairwise correlation matrix of all genes corroborates results on metabolic function of CD4^+^ T cells from differing lactation stages

Furthermore, we evaluated gene expression data using Pearson’s pairwise correlation matrix to compare glycolytic and mitochondrial function with immune and metabolic signatures (Fig. 6b). We were able to identify distinct clusters based on lactation stage. When analyzing all genes impacting immune and metabolic function, CD4^+^ T cells from early lactation cows were most dissimilar from other stages, whereas CD4^+^ T cells from late lactation and dry cows formed a cluster. It is of note, when analyzing gene expression data focusing on genes involved in metabolism, as well as signaling molecules affected by metabolic enzymes, CD4^+^ T cells isolated from mid-lactation and dry cows grouped together (Supp. Fig. S4, S5). In contrast, CD4^+^ T cell cytokine mRNA expression clustered mid and late lactation groups together, as CD4^+^ T cells from dry cows expressed the highest levels of cytokine mRNA, which paired with similar cytokine protein levels (Fig. 6c, Supp. Fig. S6). IFN-γ, IL-2, and TNF-α are produced at a higher level in stimulated CD4^+^ T cells isolated from late lactation, but most distinctly, from dry cows (Fig. 6c–e). No protein assays for genes involved in metabolism have been examined for comparison to observations of mRNA expression data. However, it is likely that post-transcriptional or post-translational modifications in CD4^+^ T cells from dry cows differ from those of mid-lactations cows, which would account for the discrepancy between the mRNA expression and observations seen from extracellular flux analysis.

## Discussion

Lactation and pregnancy are dynamic processes, modulating physiology and immunity of cattle. Our study provides novel data on the immunometabolic impact lactation has on activated CD4^+^ T cells. Bovine CD4^+^ T cells in early lactation have an overall reduced immune response in relation to CD4^+^ T cells isolated from cows at other lactation stages and dry cows. Activated CD4^+^ T cells from early lactation present the lowest mitochondrial and glycolytic function and were the lowest cytokine producers. Gene expression analyses revealed CD4^+^ T cells during early lactation have a unique gene expression signature in comparison to those cells isolated during other lactation stages and dry period. CD4^+^ T cells from dry and late lactation cows appeared to be the most metabolically active, but surprisingly, had different gene expression levels. Interestingly, activated CD4^+^ T cells from mid-lactation cows have decreased rates of both OXPHOS and glycolysis when compared to late lactation and dry cows, but had a similar metabolic gene expression profile compared to dry cows. It remains to be determined why CD4^+^ T cells from mid-lactation and dry cows exhibit functional differences, despite similar metabolic signatures. We predict translation and post-translational regulation play a role in modulating protein expression and function, thus driving two separate metabolic and functional phenotypes.

Susceptibility of dairy cattle to a variety of pathogens and metabolic disease is impacted by stage of lactation^36^. Schwarm *et al*.^15^, suggested that nutrient availability in pre- and postpartum periods (up to 5 weeks) may contribute to immunity of cattle. Blood glucose, insulin, and NEFAs are useful in determining energy balance. In the present study, serum levels of glucose and insulin were within accepted physiological ranges during all lactation stages^37,38^. NEFA levels are increased in early lactation, as our data supports (Fig. 1). Lacetera *et al*.^27^, suggested that PBMCs from cows with increased plasma NEFAs have decreased functions *in vitro*. Others found NEFAs play a role in cytokine suppression and gene regulation, or alternatively, have immunostimulatory roles in innate immune cells^39–41^. In addition, lipid mobilization during early lactation increases concentration of NEFAs but may also alter concentrations of certain fatty acids in the lipid fraction. For instance, saturated fatty acids, palmitic and stearic acid, are increased in plasma of early lactation cows, but are not found at high levels in later lactation stages or the dry period^25^. Further, Contreras *et al*^25^. showed these two saturated fatty acids incorporate into PBMC cellular membranes and suggested that changes in fatty acid composition may play a role in the immunosuppressive profile in early lactation cows. Further, linoleic acid is increased in total plasma lipids^25^, which is implicated in mitochondrial depolarization and apoptosis in CD4^+^ T cells, even in small amounts^26^.

In our study, CD4^+^ T cells from early lactation cows were cultured *ex vivo* for 24 hours in the presence of glucose and other nutrients. Early lactation cows showed reduced metabolic function and cytokine production in comparison to late lactation and dry cows. Evidently, CD4^+^ T cells isolated from early lactation cows were incapable of “recovering” functional capacity in a nutrient enriched environment *ex vivo*. In support of this diminished metabolic phenotype, fatty acid molecules and NEFAs have been shown in humans and rats to affect cellular bioenergetics, inhibiting activation of the PI3K pathway through insulin receptor signaling^42,43^. We predict compositional changes caused by an influx in NEFAs in plasma negatively impact membrane proteins involved in immune activation and energy uptake, and thereby modify bioenergetic pathways.

In terms of cellular metabolism, like activated human and mouse CD4^+^ T cells, activated bovine CD4^+^ T cells increase aerobic glycolysis and OXPHOS compared to resting CD4^+^ T cells. (Fig. 2). Increased basal OCR levels is indicative of an activated state and has been previously reported by Schwarm *et al*.^15^ in bovine PBMCs. Activation was further confirmed by an increased forward scatter (data not shown). However, CD4^+^ T cells isolated during different lactation stages exhibit variable rates at which they increase these energetic pathways (Figs. 3 and 4). Though mitochondrial parameters were not significantly different among lactation stages, higher trends in basal OCR and proton leak were observed in CD4^+^ T cells from late lactation and dry cows, in comparison to cells from cows in early and mid-lactation (Fig. 3b,c).

Proton leak (Fig. 3c), the uncoupled translocation of protons from intermembrane space (IMS) of mitochondria when ATP synthase is inhibited, as occurs during injection of oligomycin, is an important pathway for preventing oxidative damage^44^. There is evidence that reactive oxygen species induces proton leak^45^. To counteract overproduction of ROS, uncoupling proteins are employed to allow H^+^ to move across the IMS from mitochondria^46^. With that in mind, it is possible that increased proton leak may be a compensatory mechanism in dry cows, following an increased production of ROS. Despite early studies showing ROS to have cell-damaging capabilities, it has recently been shown to play a role in cell signaling^47,48^. An increase in ROS could partly explain increased cytokine production in CD4^+^ T cells isolated during late lactation and the dry period (Fig. 6c). However, this remains to be elucidated. Studies with lactating mice show similar results, in that lactating mice have varied mitochondrial respiratory function depending on stage of lactation^49^.

Maximal respiration (Fig. 3d), which is the maximal capacity the respiratory chain can operate in a physiologically energetic demanding situation, is consistent among stages, as is spare respiratory capacity (Fig. 3e). Spare respiratory capacity indicates cellular capability to respond to an increased energetic demand, as well as a measurement of cellular capability of respiring at theoretical maximum^50^. CD4^+^ T cells are operating at proportional levels to machinery available to develop an appropriate response to an energetic demand. Taken together, these data may suggest an alteration in mitochondrial metabolic phenotypes of CD4^+^ T cells, as T helper subsets have been shown to have differing metabolic phenotypes^51,52^. We realize that this study was limited in assessing the contribution of specific T helper subset to overall mitochondrial function.

Investigating further the differences in mitochondrial function, we found through mitochondrial mass analyses a 3.5-fold increase in CD4^+^ T cells isolated from dry cows which could play a role in the observed increase in OCR (Fig. 4a). Flow cytometric measurements of mitochondria mass were compared to the mtDNA/nDNA ratio, another measurement of biogenesis. While there was a slight increase mtDNA/nDNA in CD4^+^ T cells isolated from dry cows (Fig. 4b), it is unclear if the increase in mitochondrial mass is because of an increase in mitochondrial content. With this increase in mitochondrial mass, it is possible that mitochondria in CD4^+^ T cells isolated from dry cows are undergoing morphological changes, such as increased fusion events. Mitochondrial fusion is the process of two mitochondria fusing together inner and outer membranes during a stress or increased energy demand to compensate for damage and/or increase oxidative capacity, a process that is GTPase driven^53^. This corroborates increased OCR levels seen from stimulated CD4^+^ T cells from dry and late lactation cows in Fig. 4. In addition, fusion events are prevalent in memory T cells during activation, so it could be attributed to heterogenous CD4^+^ T cell populations between lactating cows of different stages or as part of a normal physiological process^30,54^.

Despite minor increases in OCR in CD4^+^ T cells isolated from different lactation groups, we found the most dramatic differences between CD4^+^ T cells from these groups in aerobic glycolysis rates (Fig. 5). Glucose is a major energy source for bovine lymphocytes^2^, and fuels aerobic glycolysis. This pathway regulates immunity by providing molecules for pathways used during cell cycle progression and activation^55,56^. Stimulated CD4^+^ T cells from all stages effectively increased their glycolysis rate compared to unstimulated cells (Fig. 5a).

Furthermore, stimulated cells from late and dry cows had a significantly higher rate of glycolysis as well as higher glycolytic capacity compared to stimulated cells from early and mid-lactation. Upregulation of aerobic glycolysis in activated cells requires an increase in cellular glucose entry. Glucose transporters, Glut1 and Glut3, in monocytes and macrophages have been shown to be modulated by milk production postpartum^57^. As identified in murine T cells, Glut1, and to a lesser extent Glut3, are important in transporting glucose into the cell, with Glut1 being essential to activation and effector function^58^. Despite mRNA expression of glycolytic machinery and glucose transporters having different levels of expression in CD4^+^ T cells from late lactation and dry cows, perhaps post-transcriptional or translational modifications are occurring in cells from these two groups, driving this increased glycolytic rate (Figs. 5b and 6a). Interestingly, glycolytic reserve (Fig. 5d), an indicator of cellular ability to utilize glycolysis during ATP demand, was consistent in activated CD4^+^ T cells across lactation stages. So, while activated CD4^+^ T cells from late and dry cows can rapidly implement glycolysis and function at a higher capacity in the event of mitochondrial dysfunction, activated CD4^+^ T cells from mid and early lactation have comparable levels of glycolytic reserve to other stages in the face of increased energetic demand. In the presence of increased energetic demand, we observed CD4^+^ T cells isolated from late lactation and dry cows have an ability to increase glycolysis even though their glycolytic reserve is like those cells isolated from cows during mid and late lactation. We attribute this rapid influx of glucose to differing levels of metabolic regulation.

Metabolism is highly regulated and it orchestrates intricate and dynamic processes, involving nutrient uptake to the regulation of bioenergetic signaling and machinery. To further understand the functional capacity of bovine CD4^+^ T cells during differing lactation stages, we examined gene expression and cytokine production. We showed that lactation groups which paired together at the protein level and shared a similar metabolic phenotype, did not necessarily exhibit similar gene expression (Fig. 6a, Supp. Fig. S3, S4, S2). A recent study of human T cells show translational machinery as a key facilitator in T cell metabolic reprogramming^51^. In corroboration with data from others^59^, our results showed that mRNA expression is a poor indicator of the changes in metabolism occurring at the protein level during T cell activation. Relying on machinery involved in translation and posttranslational modifications, could allow a more rapid response in rewiring metabolism of T cells exiting their quiescent state^52^.

Along with aerobic glycolysis and GAPDH playing a role in immunoregulation, there are other “RNA-enzyme-metabolite networks” that play a role in posttranslational modifications affecting immune function^35^. Metabolic genes, and those related to metabolism, were expressed at similar levels in CD4^+^ T cells from dry and mid-lactation cows. However, cytokine mRNA expression presented a similar signature to that observed at the protein level. In Fig. 6a, we show GAPDH is more highly expressed in CD4^+^ T cells from dry cows, and to a lesser extent in cells from mid and late lactation, with even less in cells from early lactation. GAPDH, as mentioned above, translationally regulates IFN-γ by binding to the AU-rich elements in the 3′ UTR^14^. In contrast, LDHA increases IFN-γ expression independent of 3′ UTR^60^. Both GAPDH and LDHA regulate IFN-γ during translation. T-bet, a commonly known Th1 transcription factor, promotes IFN-γ at the transcriptional level^61^. Furthermore, during activation glucose entry into the cell is increased. Because of this, HK2 (hexokinase II), the first step of glycolysis in which glucose is phosphorylated and thus trapped in the cell, is increased^62^.

With these factors working in a coordinated fashion during activation and glucose influx, mRNA of IFN-γ shows a similar expression pattern relative to its protein expression (Fig. 6c). CD4^+^ T cells from dry and late lactation cows have similar cytokine gene expression levels, which fit well with data on protein levels. It is possible mid-lactation cows have mechanisms in place to dampen glycolytic protein expression with a resultant decrease IFN-γ production. Gene expression from early lactation cows was most different from other groups. Coordination between transcription factors and signaling molecules affect T cell biology during activation. Lactation stage may impact regulation of translation and post-translational modifications. Thus, further research is required to understand impact of lactation on the complex balance between protein expression of these transcription factors and signaling molecules during CD4^+^ T cell immunometabolism.

In conclusion, changes observed in immunity of early lactation cows compared to dry cows has been attributed to physiological imbalance^41^, negative energy balance^15^, hormones^3^, and milk production^57^. Our data further suggests that altered metabolic function of activated CD4^+^ T cells isolated from early lactation cows may contribute to altered immune responsiveness of these cows to infection, as a result of reduced function of their activated CD4^+^ T cells.

## Materials and Methods

### Animals

Twenty-three healthy Holsteins from the Iowa State University Dairy Farm were selected for this study, 5–6 cows per group. All procedures were conducted in strict accordance with federal and institutional guidelines and were approved by the Iowa State University Institutional Animal Care and Use Committee (IACUC). Lactation groups were assigned as the following: Early lactation 14–43 days in milk (DIM) (n = 5), mid 81–147 DIM (n = 6), late 243–354 DIM (n = 6), and those not lactating indicated as dry cows (n = 6). Late lactation cows and dry cows were pregnant. Average days in gestation for late lactation cows and dry cows 131.667 ± 79.333 and 239.833 ± 15.167 days, respectively. Somatic cell counts and 305ME data can be found in Supplementary Fig, 1. There were no overt signs of clinical disease.

### Blood and serum collection

Blood and serum collection were performed post-milking and during feeding. 120 mL of peripheral blood was collected from each animal by jugular venipuncture using a 1:10 dilution of 2X acid citrate dextrose (ACD) in 60 mL tubes. One vacutainer tube was also taken for serum collection. Serum was centrifuged at 1173 × g for 10 minutes, aliquoted, and stored at −80 °C until further analysis.

### Serum analyses

Serum was thawed to room temperature before determining concentrations of glucose (FUJIFILM Wako Diagnostics U.S.A., Inc. #997-03001) (intra-assay CV 2.33%) and non-esterified fatty acids (NEFAs) (FUJIFILM Wako Diagnostics U.S.A., Inc. #999-34691) (intra-assay CV 0.68 ± 0.07% and inter-assay CV 2.83 ± 2.08%, as per manufacturer) by *in vitro* enzymatic colorimetric assay. Insulin (Mercodia, #10-1201-01) (intra-assay CV 3.833 ± 0.867% and inter-assay CV 7.1 ± 0.153%, as per manufacturer) was assessed, also, by using an ELISA. Assays were performed according to manufacturer’s protocols.

### PBMC isolation and CD4^+^ T cell enrichment

Peripheral blood mononuclear cells were processed using Accuspin tubes (Sigma #A2055). Peripheral blood was diluted with PBS and added to Accuspin tubes that contained Histopaque 1077 (Sigma #10771) under the frit. Cells were centrifuged at 1173 × g for 10 minutes. The buffy coat layer above the frit was added to a new 50 mL tube and was processed as previously described^63^. Briefly, cells were washed once with PBS at 422 × g 10 minutes. A lyse and restore step was used to remove residual red blood cells. Cells were washed two more times with PBS, counted and sorted for CD4^+^ T cells.

Bovine CD4^+^ T cells were sorted using a protocol similar to that which we have previously published^63^. Briefly, cells were sorted by positive selection using anti-CD4 (WSU, clone: ILA11A), MACs IgG2a+b beads (Miltenyi Biotec #130-047-201) and LS MACS columns (Miltenyi Biotec #130-042-401) according to manufacturer’s instructions. Purity of CD4^+^ T cells was >90%. Cells were added to RPMI 1640 (ThermoFisher #22400-089), 5% fetal bovine serum, and an antibiotic-antimycotic and incubated at 37 °C for 24 hours.

### Cell culture

CD4^+^ T cells were cultured in RPMI media (as described above), pH 7.45, and incubated at 37 °C in 5% CO_2_ for 24 hours. Optimal concentrations of anti-CD3 and anti-CD28 were determined by analyzing cell activation as assessed using forward and side scatter profiles and proliferation assays (data not shown). Plate-bound anti-CD3 at 5 μg/ml (WSU clone: MM1A) and soluble anti-CD28 at 1 μg/ml (WSU clone: TE1A) was selected to generate activated CD4^+^ T cells. After 24 hours, cell culture supernatants from stimulated and unstimulated cultures were centrifuged, aliquoted and stored at −80 °C until utilized for further analyses. Cell viability post sort was 82.957 ± 1.134; 73.0 ± 1.142 for 24 hr non-stimulated cultures; and 62.87 ± 1.384 for 24 hr stimulated cultures.

### Metabolic assays

Metabolic assays were performed using the XF^e^96 Seahorse extracellular flux analyzer (Agilent Technologies). Anti-CD3:anti-CD28 stimulated and unstimulated CD4^+^ T cells were plated at 4.5 × 10^5^ live cells/well in plates coated with Cell Tak (Corning #354240). Final well drug concentrations for the Mito Stress Test (Agilent #103015-100) were as follows: Oligomycin 2 μΜ, FCCP (Carbonyl cyanide-4-phenylhydrazone) 2 μM, and Rotenone/Antimycin A 0.5 μΜ. Culture media for the Mito Stress Test contained RPMI and 2 mM glutamine without a buffer solution (Sigma #R1383), 10 mM glucose (Sigma #S8636) and 1 mM pyruvate (Sigma #G8769). Equations for parameters and a graphical representation of calculations from the report generator used for Mito Stress Test can be found in Supplementary Fig. S2. Final well concentrations for the Glycolysis Stress Test (Agilent #103020-100) were as follows: Glucose 10 mM, Oligomycin 2 μM, 2-Deoxyglucose 50 mM. Culture media for the Glycolysis Stress Test was RPMI containing 2 mM glutamine without a buffer solution (Sigma #R1383) and no glucose or pyruvate. Equations for parameters and a graphical representation of calculations from the report generator used for Glycolysis Stress Test can be found in Supplementary Fig. S3.

### Flow cytometry

Cell surface staining was conducted on sorted CD4^+^ T cells. Briefly, 2 × 10^5^ cells were washed with PBS and incubated with Live/Dead stain (ThermoFisher #65-0865-14) for 30 minutes at 4 °C. Cells were washed and incubated with primary antibodies: anti-CD3 (WSU, IgG1 clone: MM1A), anti-CD4 (WSU, IgG2a, clone: IL11A), for 15 minutes at room temperature. Cells were washed and incubated in the dark for 15 minutes with the following secondary antibodies: anti-IgG1 AF350 (ThermoFisher #A21120); anti-IgG2a PE (Biolegend, clone: RMG2a-62 #407108); anti-IgG3 AF488 (ThermoFisher #A-21151), anti-IgG2b (AF350, ThermoFisher #A-21140). Cells were washed and analyzed using a Becton Dickinson LSR II flow cytometer. To determine mitochondrial mass, cells were incubated for 20 min. at room temperature in the dark with 25 nM Mitotracker green (ThermoFisher #M7514). Data were evaluated with FlowJo software (FlowJo, LLC, Ashland, OR, USA).

### Realtime PCR

To analyze mRNA expression, as well as genomic DNA for mtDNA/nDNA ratios, an Allprep RNA/DNA/Protein mini kit (Qiagen #80004) was used to extract RNA and DNA. RNA purity, accessed using A260/A280 ratio which exceeded 1.7. RNA integrity was checked on a subset of 15 samples using an Agilent 2200 TapeStation (Agilent Technoligies, Santa Clara, CA, USA), with RIN numbers ranging from 7.6 to 9.5. RNA was used to transcribe single-stranded cDNA as previously described^64^ using random primers (ThermoFisher #48190-011) and dNTP mix (Thermofisher #18427-088). 5x First Strand Buffer, 0.1 M DTT, and Superscript Reverse Transcriptase (ThermoFisher #18080-044) were added as per manufacturer’s instructions to complete the reaction. cDNA was added to a master mix of the respective primers with DNase and RNase free water as well as Power SYBR green PCR master mix. (ThermoFisher #4367659). Expression levels of mRNA were normalized to the housekeeping gene RPS9 (Ribosomal Protein S9) and analyzed by relative quantification using ∆∆CT method. Primers were previously published or designed for this study using Primer-Blast primer software (NCBI). The specificity of primers was verified by combinations of melting curve analysis, gel electrophoresis and/or sequencing. List of primers used in the present study can be found in Table 1.

**Table 1:** Primer sequences of genes used in real-time PCR.

Gene^a^			Primer sequences (5′-3′)	Accession #	Source
Signaling	*mTOR*	Fwd:	ATGTGCGAACACAGCAACAC	XM_010823084.1	This paper
		Rev:	CCTTTCACGTTCCTCTCCCC		
	*Rictor*	Fwd:	TGGCTCAATGCCTCTTCTGG	XM_010816811.1	This paper
		Rev:	TTGGAAAGGATGACCCTGGC		
	*Raptor*	Fwd:	AGCTTTGCACGTCTTTACGC	XM_010816454.1	This paper
		Rev:	GCAGCGACCTTGTTGAAGAC		
	*INSR*	Fwd:	TCCTCAAGGAGCTGGAGGAGT	XM_590552	^66^
		Rev:	GCTGCTGTCACATTCCCCA		
Glycolysis	*Glut1*	Fwd:	GTGCTCCTGGTTCTGTTCTTCA	NM_174602	^67^
		Rev:	GCCAGAAGCAATCTCATCGAA		
	*Glut3*	Fwd:	GCCGCCGATAGAGGACATTT	NM_174603.3	^57^
		Rev:	ATGGCGAAGATCAGAGGTGC		
	*HK2*	Fwd:	AAGATGCTGCCCACCTACG	XM-002691189	^68^
		Rev:	TCGCTTCCCATTCCTCACA		
	*FBP1*	Fwd:	CACCGAGTATGTCCAGAGGAAGA	Bt.24314	^69^
		Rev:	ACGTACCTGGCGCCATAGG		
	*GAPDH*	Fwd:	CCTGCCCGTTCGACAGATA	NM_001034034.1	^70^
		Rev:	GGCGACGATGTCCACTTTG		
	*LDHa*	Fwd:	GGCAAAGACTATAATGTGACAGCAA	BC146210	^71^
		Rev:	ACGTGCCCCAGCTGTGA		
	*PDHA1*	Fwd:	CAGTTTGCTACTGCTGATCCTGAA	NM_001101046	^71^
		Rev:	AGGTGGATCGTTGCAGTAAATGT		
	*PC*	Fwd:	CTCCCACCATCTGTCCTTTCC	NM_177946	^71^
		Rev:	TTTATTTGGGCAGGAGATGAATACG		
	*ACSS2*	Fwd:	ACCCAAGGGCGTGTTACACA	Bt.29433	^69^
		Rev:	TCCTCCGCATGAAAGTCAAAC		
Cytokines	*IL10*	Fwd:	TTACCTGGAGGAGGTGAT	NM_001009327.1	^72^
		Rev:	GTTCACGTGCTCCTTGAT		
	*IL13*	Fwd:	CTGCAGTGTCATCCAAAGGA	NM_174089.1	This paper
		Rev:	GAGGGCTTGTGAGGACAGAG		
	*IL4*	Fwd:	GCGGACTTGACAGGAATCTC	NM_173921.2	^72^
		Rev:	GCGTACTTGTGCTCGTCTTG		
	*IFNg*	Fwd:	AGAATCTCTTTCGAGGCCGGAG	NM_174086.1	^72^
		Rev:	TATTGCAGGCAGGAGGACCATTAC		
TFs^b^	*GATA3*	Fwd:	AACCGGGCATTACCTGTGTA	NM_001076804.1	^73^
		Rev:	AGGACGTACCTGCCCTTCTT		
	*TBET*	Fwd:	CCTGGACCCAACTGTCAACT	NM_001192140.1	^73^
		Rev:	GGTAGAAACGGCTGGAGATG		
mtDNA	*COX1*	Fwd:	GTTTCATCGTATGAGCCCACCA	NC_006853.1	^74^
		Rev:	AGTGGCTGATGTGAAGTAGGC		
nDNA	*mtTFA*	Fwd:	CAAATGATGGAAGTTGGACG	NM_001034016.2	^74^
		Rev:	AGCTTCCGGTATTGAGACC		
HKG^c^	*RPS9*	Fwd:	GTGAGGTCTGGAGGGTCAAA	NM_001101152.2	^75^
		Rev:	GGGCATTACCTTCGAACAGA		

^a^Gene names: *MTOR* mammalian/mechanistic target of rapamycin, *RICTOR* RPTOR Independent Companion of MTOR Complex 2, *RPTOR* Regulatory-associated protein of mTOR, *INSR* Insulin receptor, *GLUT1* SLC2A1 glucose transporter 1, *GLUT3* SLCA23 glucose transporter 3, *HK2* hexokinase II, *FBP1* Fructose-1,6-bisphosphatase 1, *GAPDH* Glyceraldehyde 3-phosphate dehydrogenase, *LDHA* Lactate dehydrogenase isoform a, *PDHA1* Pyruvate dehydrogenase lipoamide α 1, *PC* pyruvate carboxylase, *ACSS2* Acyl-CoA synthetase short-chain family member 2, *IFNG* Interferon gamma, *GATA3* Gata binding protein 3, *COX1* Cytochrome c oxidase subunit 1, *TFAM* Transcription factor A, mitochondrial, *RPS9* Ribosomal protein S9.

^b^TFs: Transcription factors.

^c^HKG: housekeeping gene.

### ELISAs

Concentrations of cytokines in cell culture supernatants were determined using sandwich ELISAs. Samples were thawed on ice, diluted 1:3 and were analyzed for TNF-α (Kingfisher Biotech, Inc. #VS0285B-002) and IL-2 (Kingfisher Biotech,Inc. #DIY110B-003), or diluted 1:10 for IFN-γ (Kingfisher Biotech, Inc. #VS0257B-002). Instructions as per the manufacturer were followed as indicated for TNF-α and IFN-γ. For IL-2, plates were coated with IL-2 capture antibody (2.5 μg/ml). Plates were washed and blocked for 1.5 hrs and samples and standards were loaded and incubated for 1 hr. Plates were washed and detection antibody (0.1 μg/ml) was added for 1 hr. Plates were washed and streptavidin-HRP was added and incubated for 30 min. Following washing, plates were developed for 30 min with TMB substrate solution and reactions were stopped with Stop Solution (Kingfisher Biotech, Inc. #AR0133-002) and read on a Flex Station 3 microplate instrument (Molecular Devices, San Jose, CA, USA) at 450 nm. TNF-α (KingFisher Biotech, Inc. #VS0285B-002) inter-assay CV 17.028 ± 10.848%. IL-2 (KingFisher Biotech,Inc. #DIY110B-003) inter-assay CV 12.901 ± 10.676%. IFN-γ (Kingfisher Biotech, Inc. #VS0257B-002) inter-assay CV 6.1 ± 0.213%.

### Statistical analyses

Sample size was determined using power analysis. The effect size on which our calculations were based was estimated from results of previous studies using OCR/ECAR ratio of non-stimulated and stimulated CD4^+^ T cells. With an α = 0.05 and power = 0.80, the projected sample size needed to measure a significant effect between groups would be n = 4 animals/group, calculated using G*Power 3.1.9 Software (http://www.psycho.uni-duesseldorf.de/abteilungen/aap/gpower3/). As stated in an earlier section, lactation group size exceeded n = 5 animals/group. We performed statistical analyses with GraphPad Prism8 (GraphPad Software Inc., San Diego, CA) using linear models fitting lactation stage (early, mid, late, dry) and/or treatment (resting or activated) as fixed effects. No random effects were fit and there were no correlations between fixed effects. Normality was checked by residuals, Spearman’s test for heteroscadacity and QQ plots. For non-parametric analysis, R (R Core Team, 2014) was used for statistical analysis. All data are presented as mean ± SEM. Statistical significance is indicated by *p < 0.05, **p < 0.01, and ***p < 0.001.

### Serum analyses

Glucose, NEFAs, and insulin concentration were analyzed using lactation stage (early, mid, late, dry) as the fixed effects by ordinary one-way ANOVA and Tukey’s multiple comparison test, post-hoc.

### OCR/ECAR ratio analysis

Basal energy metabolism was assessed by OCR/ECAR ratios and log-transformed for statistical analyses. Stage of lactation (early, mid, late, dry) and treatment (resting, activated) were fitted as fixed effects for measurement of metabolic output (OCR and ECAR) and analyzed for significance using an ordinary two-way ANOVA and Sidak’s multiple comparison test post-hoc.

### Mitochondrial and glycolytic stress tests

Mitochondrial and glycolytic function of activated CD4^+^ T cells was assessed using lactation stage (early, mid, late, dry) as the fixed effect. Resting CD4^+^ T cells were used as a point of reference and were not included in the analysis. Metabolic function was assessed by OCR and ECAR values in activated cells by Kruskal-Willis and Dunn test for multiple comparisons, post-hoc. Adjusted p-values were reported using Benjamini-Hochberg multiple comparison correction and false discovery rate (FDR).

### Mitochondrial analysis

For analysis of mitochondrial mass, the ratio of geomean fluorescent intensity ratio from activated to resting state was calculated (geoMFI activated/geoMFI resting). Lactation stage (early, mid, late, dry) was the fixed effect in a one-way ANOVA with Tukey’s multiple comparison test. Changes in CD4^+^ T cell mitochondrial biogenesis, as measured by mtDNA/nDNA, was normalized for each cow [activated mtDNA/nDNA]/ (resting mtDNA/nDNA] and analyzed for differences. Lactation stage (early, mid, late, dry) was the fixed effect in the Brown-Forsythe ANOVA with Dunnett T3 multiple comparison test.

### Cytokine ELISAs

Supernatants from activated and resting cells were used to measure cytokine concentrations. Resting cells were below the level of detection in all ELISAs. Data presented are baseline-subtraction corrected to unstimulated cell supernatants and analyzed with lactation stage (early, mid, late, dry) as the fixed effect in a one-way ANOVA with Tukey’s multiple comparison.

### Real-time PCR analysis

mRNA expression was determined by ΔΔCT method, log-transformed, and checked for normal distribution. Data are represented in a heatmap with hierarchical clustering using Pearson’s distant measurement and average linkage clustering to determine gene expression patterns between lactation groups (early, mid, late, dry). Pearson’s correlation coefficient matrices were used to determine possible similarities among lactation groups and these data are represented by a heatmap, as well. Heatmaps were created using Heatmapper, a web-enabled heatmapping tool, (http://www.heatmapper.ca)^65^.

## Supplementary information

Supplementaryinformation
