# Supplementary material for: Lactation stage impacts the glycolytic function of bovine CD4+ T cells during ex vivo activation
Source: Sci Rep. 2020 Mar 4;10:4045. doi: 10.1038/s41598-020-60691-2 (PMC7055328; doi:10.1038/s41598-020-60691-2)
Supplement: Supplementary file 1 — Supplementaryinformation [file 41598_2020_60691_MOESM1_ESM.docx]

**Supplementary Information:**

**Lactation stage impacts the glycolytic function of bovine CD4^+^ T cells during *ex vivo* activation**

Jordan M. Eder^a^, Patrick J. Gorden^b^, John D. Lippolis^c^, Timothy A. Reinhardt^c^, Randy E. Sacco^a,c,*^

^a^ *Immunobiology Interdepartmental Graduate Program, Iowa State University, Ames, IA*

^b^ *Veterinary Diagnostic and Production Animal Medicine, Iowa State University, Ames, IA*

^c^ *Ruminant Disease and Immunology Research Unit, National Animal Disease Center, USDA, Agriculture Research Service, Ames, IA*


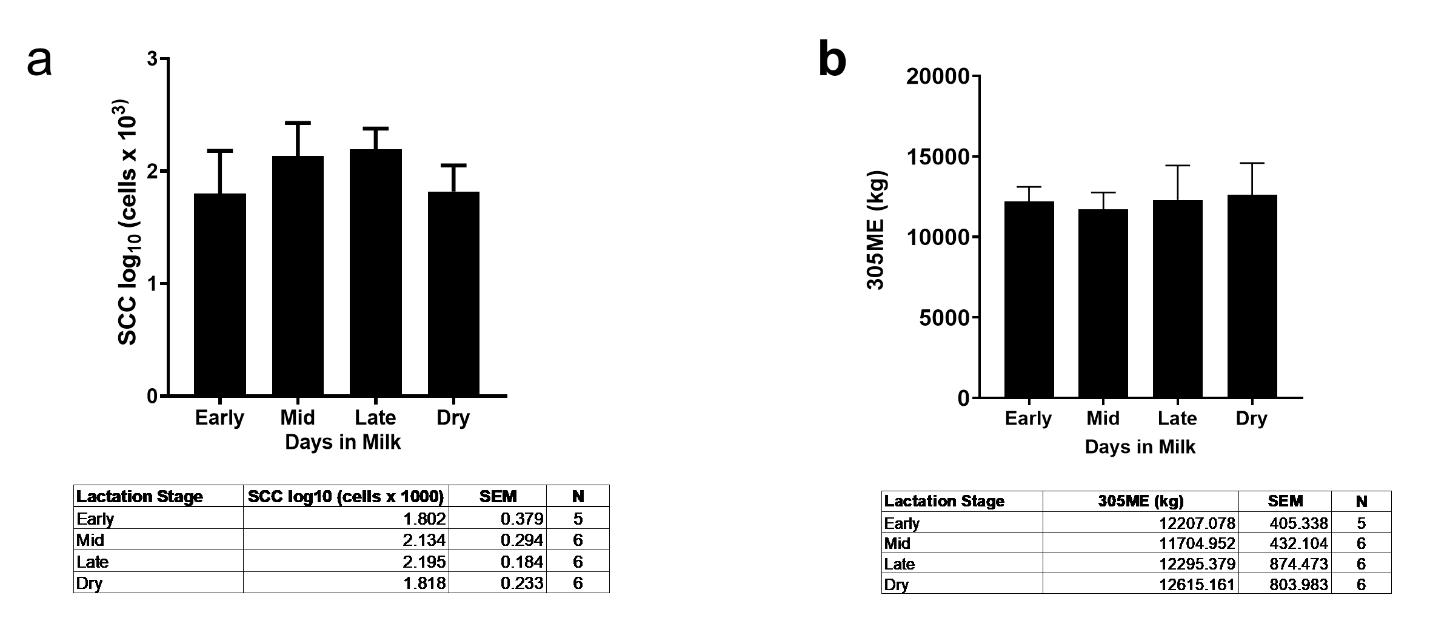


**Supplementary Figure S1. Somatic cell counts and 305ME production data from dairy cows.**

Cows were separated into groups according to lactation stage as determined by days in milk m(DIM) or indicated as dry for those not lactating. Early lactation cows (n=5) were 14-43 DIM, mid lactation cows (n=6) were 81-147 DIM, late lactation cows (n=6) were 243-354 DIM, and dry cows are not lactating. Somatic cell counts and 305 day milk yield (305ME) production data shown are mean and SEM. One-way ANOVA with Brown Forsythe and Welch tests were used for multiple comparisons among all stages.


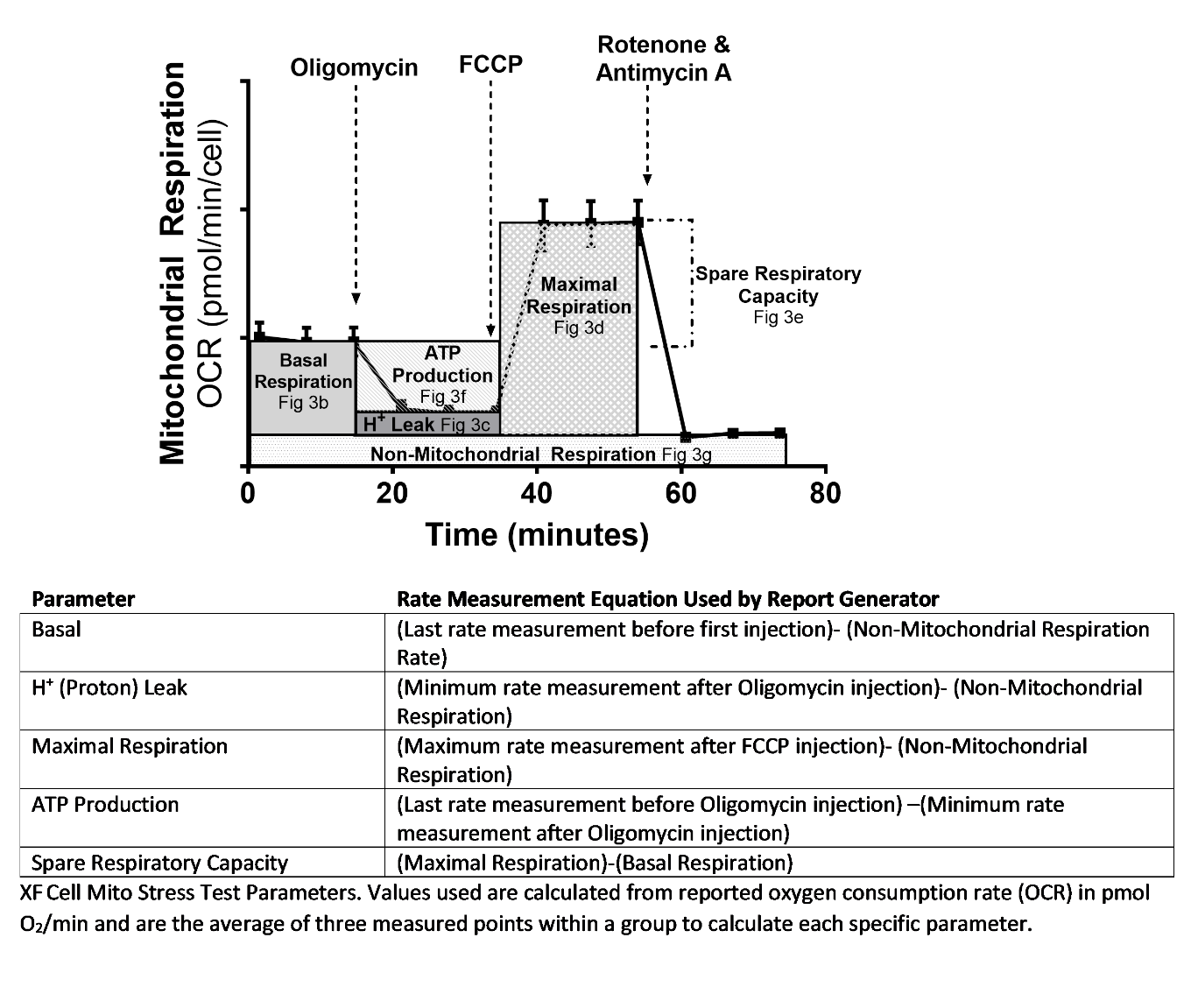


**Supplementary Figure S2. Graphical Representation of XF Cell Mito Stress Test Parameters and calculations**


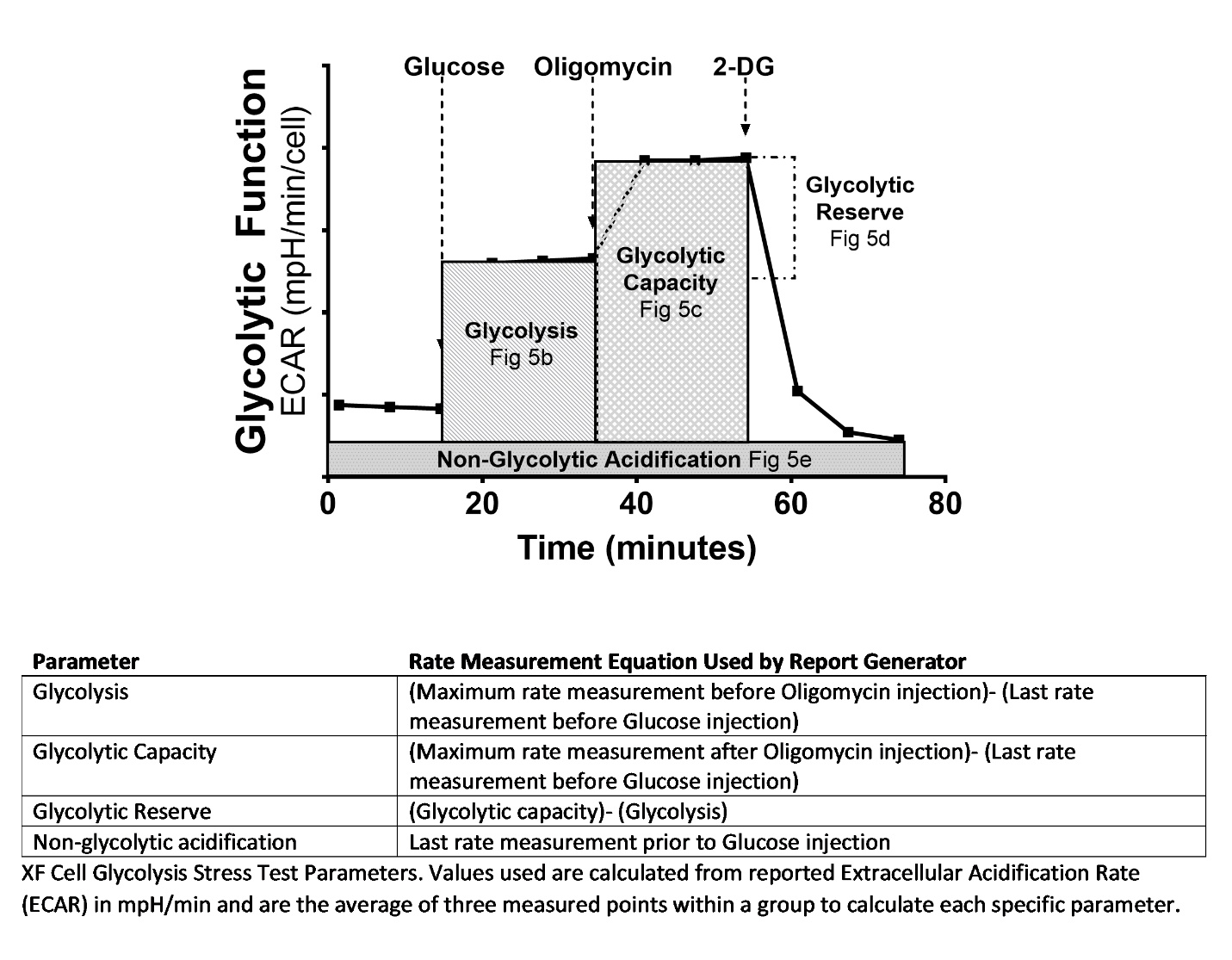


**Supplementary Figure S3. Graphical representation of XF Cell Glycolysis Stress Test Parameters and calculations**

**
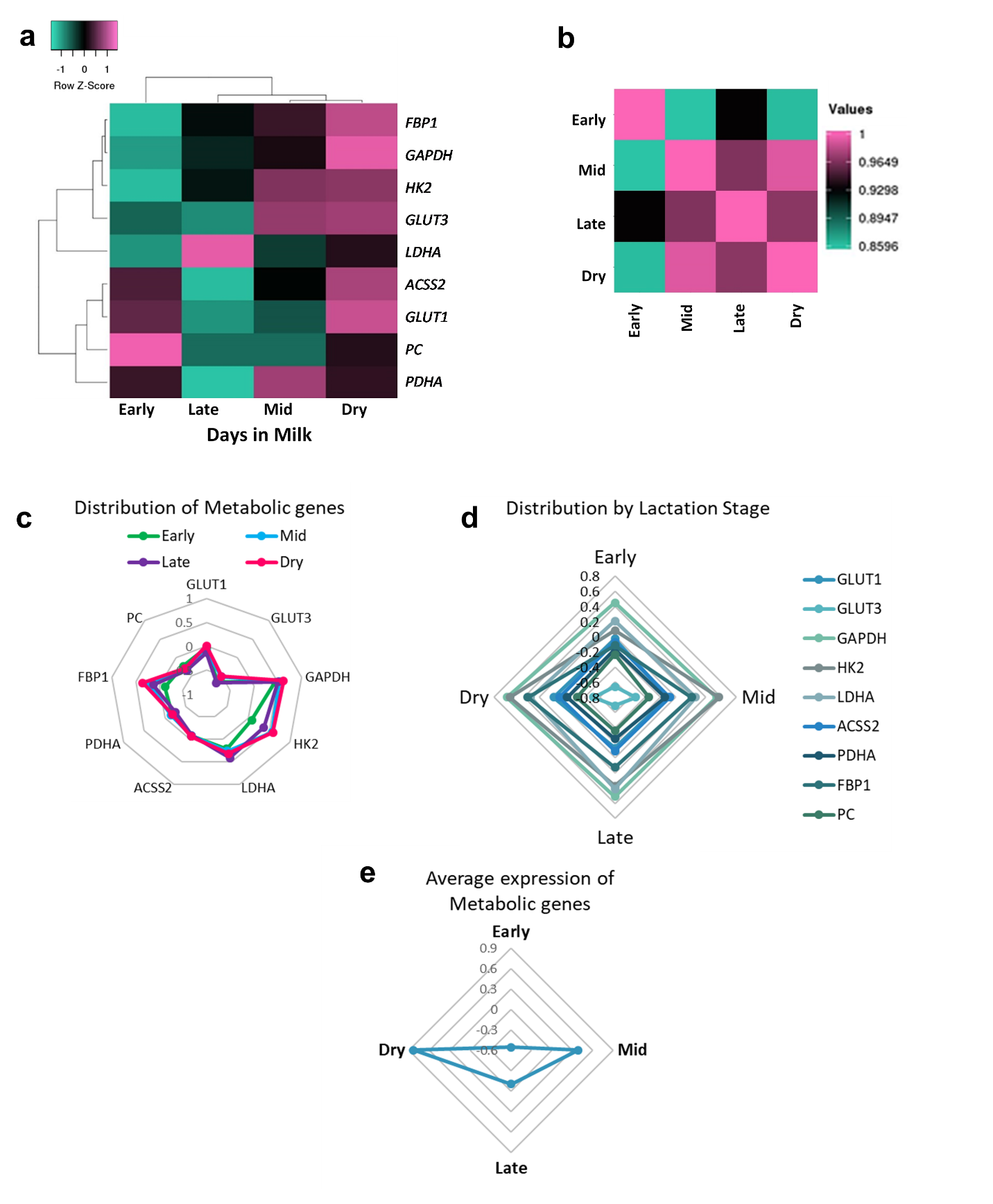
**

**Supplementary Figure S4**. **mRNA expression patterns of metabolic-associated molecules in CD4^+^ T cells from lactating and dry cows.** mRNA expression of enzymes and receptors associated with metabolic activities were analyzed independently and hierarchically clustered by average linking and Pearson’s distance measurement by the log­_2_ relative expression from qPCR (A). (B) Pairwise Pearson’s correlation plot of the lactation groups (Pearson correlation coefficient 0.7059-1 for all lactation stages). (C) Radar plots of the magnitude of gene expression by lactation stage. (D) Radar plot of the distrubution of individual gene expression calculated for metabolism. The length of a spoke is proportional to the magnitude of that particular gene in each stage of lactation (E) Radar plot of the distrubution of gene expression calculated by metabolism. In (E) the length of a spoke is proportional to the magnitude of metabolic pathways. Dry and mid lactation have the highest levels of mRNA encoding for genes involved in metabolism


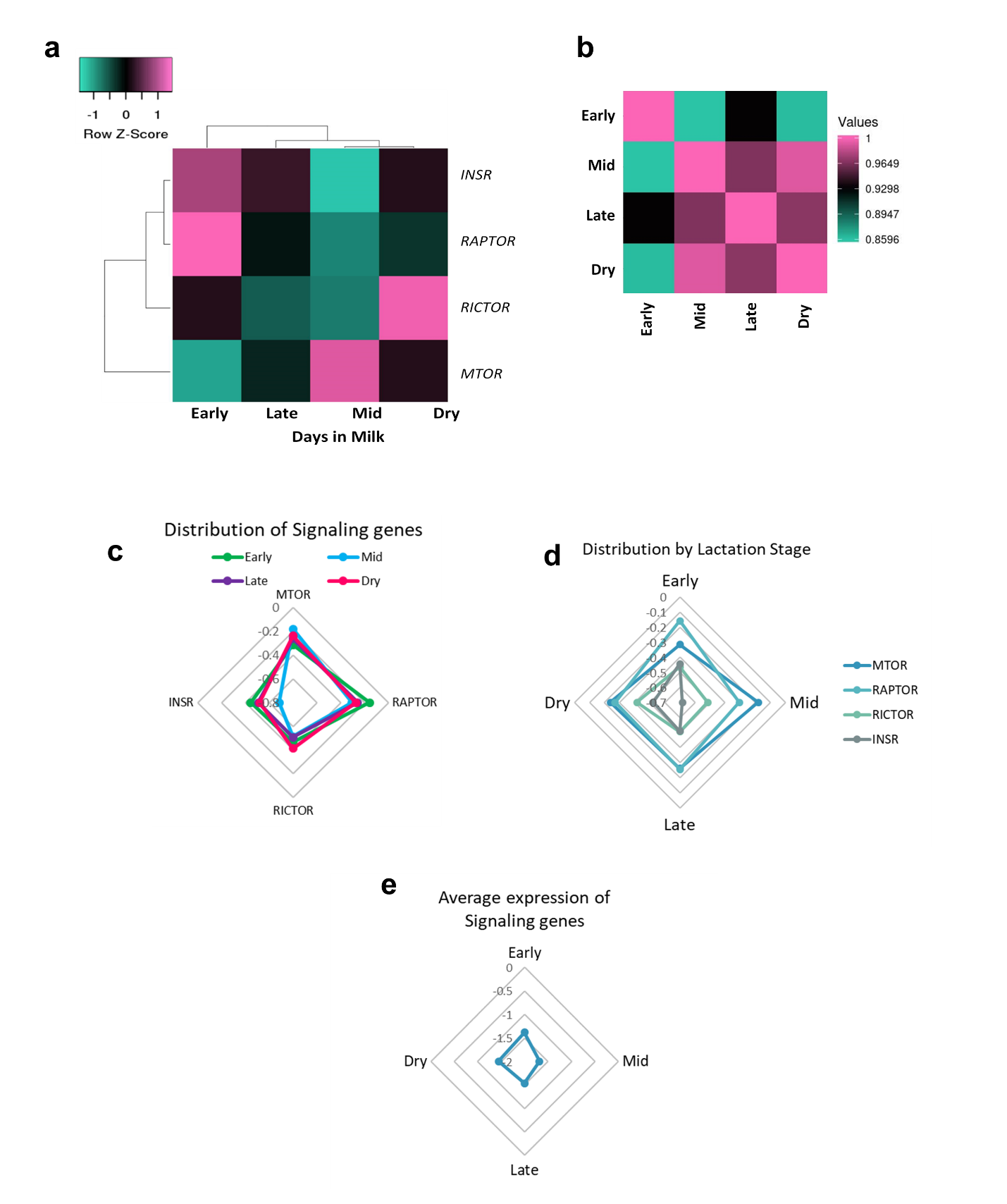


**S****upplementary Figure S5**. **mRNA expression patterns of intracellular signaling in CD4^+^ T cells from lactating and dry cows** mRNA expression of proteins involved in signaling associated with metabolic activities were analyzed independently and hierarchically clustered by average linking and Pearson’s distance measurement by the log­_2_ relative expression from qPCR (A). (B) Pairwise Pearson’s correlation plot of the lactation groups (Pearson correlation coefficient 0.5159-1 for all lactation stages). (C) Radar plots of the magnitude of gene expression by lactation stage. (D) Radar plot of the distrubution of individual gene expression calculated for metabolism. The length of a spoke is proportional to the magnitude of that particular gene in each stage of lactation (E) Radar plot of the distrubution of gene expression calculated by metabolism. In (E) the length of a spoke is proportional to the magnitude of metabolic pathways. Dry and mid lactation have the highest levels of mRNA encoding for genes involved in signaling associated with metabolism.

**
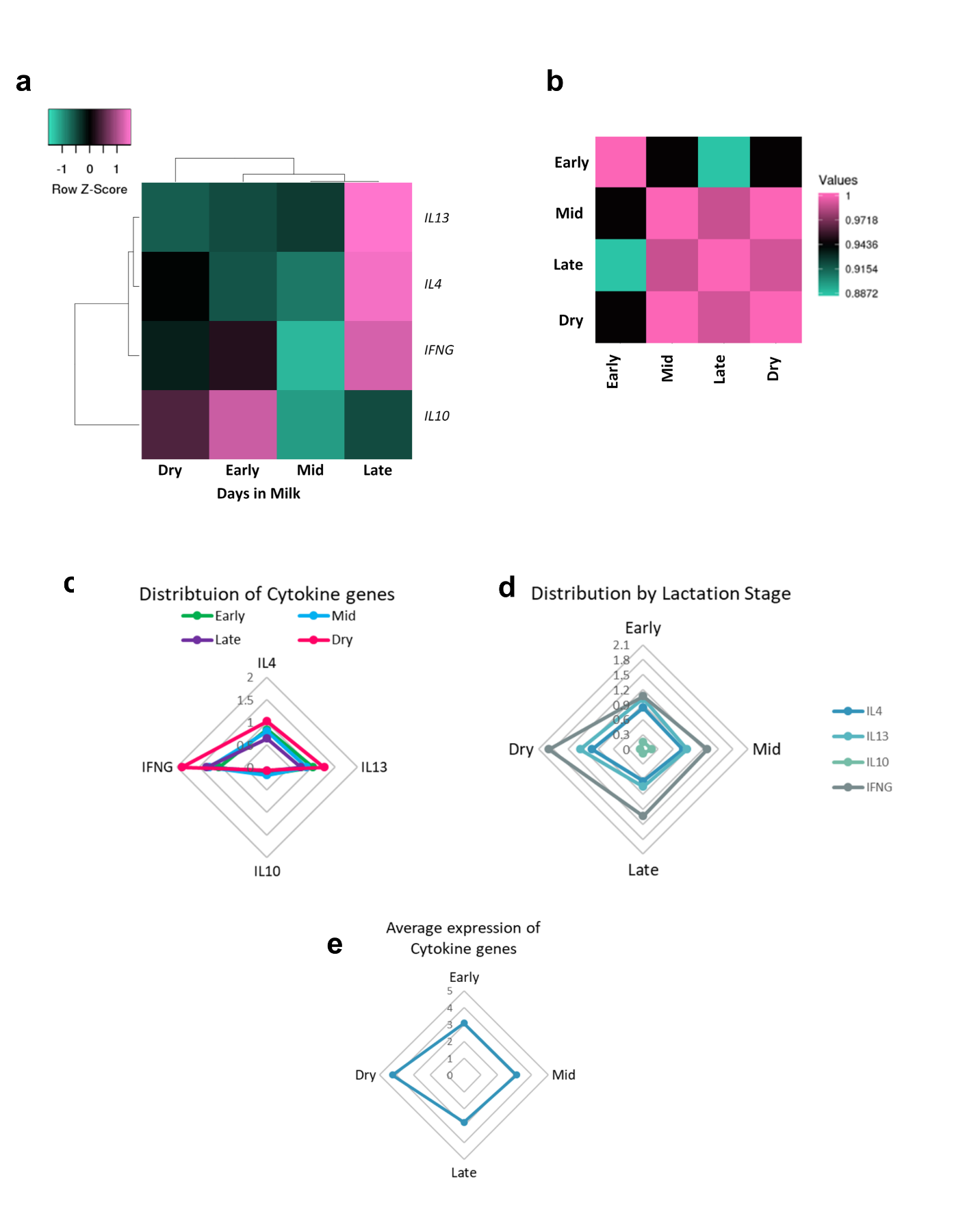
**

**Supplementary Figure S6**. **mRNA expression patterns of cytokines in CD4^+^ T cells from lactating and dry cows** mRNA expression of cytokines were analyzed independently and hierarchically clustered by average linking and Pearson’s distance measurement by the log­_2_ relative expression from qPCR (A). (B) Pairwise Pearson’s correlation plot of the lactation groups (Pearson correlation coefficient 0.8423-1 for all lactation stages (C) Radar plots of the magnitude of gene expression by lactation stage. (D) Radar plot of the distrubution of individual gene expression calculated for cytokine production. The length of a spoke is proportional to the magnitude of that particular gene in each stage of lactation (E) Radar plot of the distrubution of gene expression calculated by metabolism. In (E) the length of a spoke is proportional to the magnitude of cytokine production. Dry and late lactation have the highest levels of mRNA encoding for genes involved in signaling associated with metabolism.
